# A Protocol for Integrating Neuroscience Into Studies of Family-Based Treatment for Anorexia Nervosa: An Approach to Research and Potential Benefits for Clinical Care

**DOI:** 10.3389/fpsyt.2019.00919

**Published:** 2019-12-18

**Authors:** Cara Bohon, Noam Weinbach, James Lock

**Affiliations:** ^1^ Department of Psychiatry and Behavioral Sciences, Stanford University, Stanford, CA, United States; ^2^ Department Psychology, University of Haifa, Haifa, Israel

**Keywords:** neuroimaging, treatment, anorexia nervosa, family-based treatment, adolescents

## Abstract

Anorexia nervosa (AN) is a life-threatening disorder with peak onset during adolescence. Prior research supports the effectiveness of family-based treatment (FBT) for AN in adolescents, but studies do not regularly include neuroimaging to investigate the effects of FBT on the brain. This is important because we know that malnutrition has a detrimental impact on brain volume, cortical thickness, and function, which often recover with weight restoration. Additionally, early weight gain in FBT has emerged as a robust predictor of treatment outcome, yet it is unclear whether it is associated with neural change. Understanding neural change during treatment, particularly in the early weeks, has the potential to improve outcome by enhancing motivation for rapid behavior change, while also highlighting mechanisms by which early treatment response leads to improved outcome. This manuscript describes a study protocol and discusses both challenges and implications for this type of integrative research.

## Introduction

### Background and Rationale for Studying Early Neural Change in Adolescent AN

Family-based treatment (FBT) for adolescent anorexia nervosa (AN) results in clinically significant improvement in about 75% of patients and remission in between 22%–49% by the end of treatment (EOT) (1). Despite this, these numbers demonstrate that a percentage of patients do not achieve optimal outcome. Prior research has identified early weight gain of 2.4 kg by session 4 in FBT as a surrogate early marker for full weight restoration [>94% expected body weight (EBW)] by end of treatment (EOT) (2–6). An improved understanding of the neural underpinnings of early weight gain could be helpful in identifying treatment mechanisms and developing novel ways to improve FBT through behavioral reinforcement or support of refeeding effects, which will ultimately result in higher rates of recovery and decreased use of higher cost and potentially ineffective programs.

Existing randomized controlled trials (RCTs) have understandably focused attention on clinical outcomes such as weight gain and behavioral and attitudinal changes in the patients and their families (7). However, adding neuroimaging to an RCT would allow us to examine neural change over the course of treatment with particular emphasis on early neural changes. Determining neural correlates of the robust marker of early weight gain can potentially help us understand why it results in improved outcome and will allow us to further enhance treatment for those who do not show this early response. For example, the predictive effect of early treatment response may simply reflect that most change occurs early in treatment or it could be that early changes lead to neural changes that make further treatment more effective. Parents who understand the impact of their early efforts in refeeding on their children’s brains may be more motivated to make necessary changes early in treatment. Given anecdotal evidence that many parents are resistant to pushing rapid weight gain to reduce conflict during meals or make meals easier, this could be especially helpful. Further, clinically, we see many parents concerned about a focus on feeding while seemingly ignoring cognitive symptoms. Providing evidence of brain effects of early refeeding efforts can assuage this concern and motivate parental feeding.

### Prior Evidence of Change Neural Differences in AN With Recovery

There are currently no studies of brain changes over the course of FBT and no studies of brain change examining early weight change. Most studies that have investigated structural and functional brain changes with treatment have focused on weight restoration in hospital settings (8, 9). A recent study examined change in cortical thickness with short-term weight recovery and found significant change with improvements in nutrition (10). Because early weight gain in FBT is likely a consequence of consistent nutrition *via* refeeding, there may be significant brain change in early responders that influence later treatment outcome.

#### Structural

In addition to structural improvements such as brain volume (9) and cortical thickness (10), diffusion tensor imaging (DTI) studies suggest that dysfunction in brain networks in AN may be relate to reduction of myelin that surrounds the axons of neurons (11). Myelin is made of fatty acids, and thus, severe dietary (especially fat) restriction may result in deterioration of the myelin. As nutrition improves, myelination may also improve, impacting white matter integrity. Seitz and colleagues have recently conducted a meta-analysis on white matter volume in AN and found that reductions in white matter volume appear to normalize with recovery (9). Importantly, white matter volume loss predicted BMI at 1-year follow-up after hospitalization, suggesting that this is a marker for longer-term risk (12). Thus, in addition to examining change in white matter integrity during treatment, it would be useful to also investigate whether it predicts treatment response.

#### Intrinsic Connectivity Networks (Resting-State fMRI)

As a consequence of the shifts in white matter integrity, another likely area of change with recovery relates to network connectivity in the brain. Resting-state functional magnetic resonance imaging (r-fMRI) is a tool that allows us to understand how brain activity is organized into coherent and temporally correlated networks of brain regions (13). These networks, often referred to as intrinsic connectivity networks (ICNs), are consistent, have high test-retest reliability (14) and correspond with structural pathways (15). Various differences in ICN activity and connectivity relate to psychopathology and mood states (16). Two such ICNs have often been identified in psychopathology research, including in AN. The default mode network (DMN) was one of the first ICNs identified and its activity correlates with baseline non-task activation and is often implicated in self-referential processes (17). The salience network (SN) is involved in integrating information between a number of systems and involves connections between the dorsal anterior cingulate cortex and frontoinsular brain regions, as well as amygdala and ventral tegmental area, important for emotion and reward detection (16). Given the symptom-related features of self-relevant thoughts (related to body shape/weight, general self-esteem, etc.), as well as processing internal and external stimuli to make eating decisions, these ICNs seem particularly relevant in AN. Indeed, McFadden and colleagues found reduced SN and DMN activity in women with AN (18). Individuals recovered from AN showed DMN and SN activity somewhere between acutely ill and healthy controls, with a significant difference remaining between recovered and control groups in SN activity (18). However, the timeline of these changes from acute illness to recovery are unknown. Network activity can reflect ongoing brain processes at rest dispersed across a network, whereas functional connectivity, the synchronous activity between regions in a network, can reflect longer term activity such that those regions may have stronger connections due to frequent co-activation (19). When looking at functional connectivity rather than overall activity of the network, there is evidence of increased connectivity in the DMN in AN (20, 21). Although the activity across the DMN may be reduced in AN at rest, this may reflect difficulty with ceasing engagement of task-like processing (perhaps related to perseverative thinking about food, weight, etc.). However, connectivity between regions within the DMN may be strengthened due to frequent co-activation outside of rest, such as during self-referential processing throughout the day. No studies to date have looked at change in these networks over the course of treatment, but there is evidence that psychotherapeutic interventions can result in change in functional connectivity in brain networks in other patient populations (22, 23). It is also possible to utilize functional connectivity in these networks to predict treatment outcome, as connectivity in the DMN has been a consistent neural marker and predictor of treatment response in depression (24, 25).

#### Task-Based Neural Function: Set-Shifting and Fear Response to Food

Furthermore, numerous studies have investigated task-based neural activity in AN, which may relate to the illness. These are studies that have utilized fMRI to measure brain activity during specific tasks to understand neural correlates of cognitive and emotional processes. Research suggests that patients with AN have difficulty with set shifting or task switching (26). Patients often perform slower on Trail B of the Trail Making Test, make more perseverative errors on the Wisconsin Card Sorting Test, and make more errors on the Brixton Task (27), although results are not always consistent. Neuroimaging studies suggest that these difficulties may reflect neural processing differences in the prefrontal cortex and precuneus (28–30). A recent study showed task shifting deficits in acutely ill patients, but not those recovered from AN (31). Indeed, longitudinal studies show that many of these deficits improve with weight restoration (32), and performance on set-shifting tasks improved with cognitive remediation therapy (33), but it is unclear how this may change in the context of FBT and relate to early treatment response. Importantly, a recent study of adolescents with AN showed that neural differences during set shifting when acutely ill were no longer present after 6 months and renutrition (34). Based on this literature of improvements with recovery, we predict change with FBT for both patients with early treatment response or those who make gains over the full course of treatment.

In addition to set shifting deficits, there is evidence of greater activation in the amygdala in response to images of high-calorie beverages and high-calorie foods (35–37). In anxiety disorders, pre-treatment amygdala activity predicts measures of post-treatment improvement in children and adults (38, 39), and amygdala hyperfunction normalizes after exposure-based treatment in specific phobia (40). Considering that successful refeeding represents repeated exposure to feared stimuli (food) in AN, amygdala response to food images may normalize in those who respond to treatment.

#### Behavioral Function: Inhibited Risk-Taking

Harm avoidance is frequently associated with AN (41). This may drive food restriction due to avoidance of potential weight gain, which patients perceive as negative. This harm avoidance may reduce risk-taking (42), which has been found in prior research in individuals with AN (43). Although most prior research on risk-taking in AN has used the Iowa Gambling Task, evidence suggests that it is not a reliable measure of risk-taking (44). Thus, other measures, such as the Balloon Analogue Risk Task used previously in AN (43) or the Angling Risk Task (45) may provide stronger evidence of inhibited risk-taking in AN (45, 46). We will explore risk-taking using the Angling Risk Task outside of the scanner with plans to obtain neuroimaging correlates in the future if the behavioral data support the initial hypothesis of change with weight gain during treatment.

### Specific Aims and Hypotheses

Overall, the study aims to examine the neural correlates of early weight gain in adolescents with AN undergoing FBT with MRI scans conducted at baseline prior to initiating treatment, after session 4 (the point at which weight gain is predictive of clinical EOT response), and again at EOT (18 sessions of treatment, approximately 6 months with a maximum of 9 months). Although this study is designed to provide evidence of feasibility in conducting neuroimaging in the context of FBT and establish preliminary effect sizes to guide future research, we provide the following specific hypotheses that we hope to explore in this pilot study. We hypothesize greater increases in brain volumes, surface area, and cortical thickness from pre-treatment to session 4 in those who gain more than 2.4 kg during that time. We also hypothesize that network connectivity and activity during r-fMRI will show greater changes in adolescents with early weight gain compared to those who do not show early weight gain. Specifically, we predict greater increases in default mode activity and reductions in SN activity from pre-treatment to session 4 in those who gain more than 2.4 kg during that time (47). We also hypothesize improvements in white matter integrity, measured with DTI, in those who gain more than 2.4 kg by session 4 compared to those who do not, providing evidence that consistent nutritional improvements can have rapid impact on brain connectivity (48). We also hypothesize improvements in functional tasks related to cognitive and emotional functioning in those who gain more than 2.4 kg by session 4. Specifically, we expect improvements in a task-switching task as evidenced by improved behavioral performance and altered brain activity in prefrontal cortex and superior parietal cortex. During a food image task, we expect to find reduced amygdala activity in response to food versus non-food images over time in those who show early weight gain. Although the primary aim relates to neural changes at session 4 (the time where early weight gain is predictive of outcome), a secondary aim is to examine these measures at EOT, which will allow us to capture any neural changes that may be more delayed, requiring longer length of time or more weight gain for recovery. This secondary aim is in place due to the preliminary nature of the protocol, since there is no prior research related to early treatment effects. In other words, this aim increases the likelihood to detect effects given that prior research on weight restoration in AN has shown improvements across these domains.

## Methods

### Participants

Participants for this study will be recruited directly from an existing RCT. The RCT is testing an adaptation to standard FBT wherein adolescents who do not gain 2.4 kg by session 4 are randomly assigned to either continue standard FBT or to receive Intensive Parent Coaching (IPC) which involves an additional intense scene during session 4 to increase urgency to act in the parents, a parent-only session to further support parental behavior change for refeeding, and an additional family meal for greater support and guidance of parent refeeding behaviors. Prior research informed the selection of 2.4 kg as the marker of early treatment response. Five studies identified early weight gain in FBT as a possible marker of early treatment response (2–4, 6, 49) converging on a consistent weight gain range of 2–3 kg by session 4. Unpublished data on early response from the largest multi-site study of FBT for adolescent AN (2) matched the weighted average of the current published studies, a weight gain of 2.4 kg by session 4. An additional study by Hughes and colleagues (50) found that 2.8 kg by session 5 was the most sensitive predictor of remission. However, this study involved twice weekly sessions for the first 2 weeks with session 5 occurring on week 3 of treatment (unlike 1 session per week in other trials). This increased intensity of treatment delivery may have shifted the timing and amount of early weight gain predictive of remission by end of treatment (50). Thus, the proposal settled on 2.4 kg by session 4 as the marker of early response. Importantly, we considered the use of change in percentage ideal body weight or BMI-SDS (body mass index standard deviation score) per age, but the prior research showed these predictive effects with the actual weight gained regardless of initial weight, height, age, or sex.

Because participants will be recruited for the neuroimaging study prior to the start of treatment, we cannot balance the numbers of participants in a particular treatment arm. All study participants who meet eligibility criteria and are able to visit Stanford University for three imaging appointments will be invited to participate. Although the main RCT is being conducted at two sites (Stanford University and University of California, San Francisco), they are both located in the San Francisco Bay Area, and thus, are both potential sites for the recruitment of participants for this neuroimaging component. The population of the Bay Area is large and diverse, and the RCT makes specific efforts to recruit minorities for the study. The target sample size for this preliminary study is 20 in order to establish feasibility of including neuroimaging to an RCT of FBT and obtain preliminary effect sizes. Inclusion criteria include: 12–18 years of age that meet DSM-5 criteria for AN and participating in the RCT testing IPC for early non-response to FBT. Exclusion criteria include: 1) associated physical illness that necessitates hospitalization; 2) psychotic illness or other mental illness requiring hospitalization; 3) current dependence on drugs or alcohol; 4) physical conditions (e.g., diabetes mellitus, pregnancy) known to influence eating or weight; 5) Wechsler Abbreviated Scale of Intelligence (WASI) scores below the low average range defined by the test (<80); 6) family history or current child abuse or neglect (when reported in response to a question on phone screen, the perpetrator of abuse is excluded from family treatment, but the adolescent may participate with the rest of the family); 7) previous FBT for AN; 8) contraindications for MRI (e.g., orthodontia, metallic implants); 9) current or past major neurological (e.g., seizure disorder, psychosis, head trauma) or major sensory deficit. Participants are withdrawn from the study if any of the following occur: 1) sexual or physical abuse by a family member; 2) hospitalization for >21 days; 3) missing >4 consecutive appointments; worsening of psychiatric conditions such that participant would be clinically better served by referral for other treatment; 4) participant undertaking other psychotherapies during the treatment study. Visual acuity is determined by self-report, and participants with corrected vision use MR-safe lenses during scans.

### Procedure

Assessments for the main RCT will be conducted at six major timepoints (baseline, 3-month mid-treatment, EOT (maximum of 9 months), and at 6- and 12-month follow-up). These assessments will include measures of weight, parental measures, and other psychopathology variables. Weekly assessment of weight and parent factors will also be conducted. For the neuroimaging study, we invite participants to have MRI and fMRI scans at baseline, session 4 mid-treatment, and EOT. All assessors are trained to reliability in the Eating Disorder Examination (EDE) (51) and other clinical diagnostic interviews, including the Kiddie Schedule for Affective Disorders and Schizophrenia (K-SADS) (52).

Neuroimaging sessions consist of structural, diffusion-weighted, and functional scans (one resting-state and two task-based). We use a 3T GE signa scanner to collect functional and anatomical imaging data. Visual stimuli are presented with a digital projector/reverse screen display system. Echo spiral imaging is used to measure the blood oxygen level dependent (BOLD) signal as an indication of cerebral brain activation. Images consist of 31 axial slices (4.0 mm with 0.5 mm gap) covering the whole brain (RT = 2 s, echo time = 30 ms, flip angle = 80, interleave = 1, FOV = 22 cm, matrix = 64 × 64). To reduce blurring and signal loss from field inhomogeneities, an automated high-order shimming method based on spiral acquisitions is used before acquisition of functional scans. R-fMRI scans consist of 240 volumes acquired over 8 min. Functional scans during the task-based paradigms will be acquired over approximately 22 min (task switching) and 8 min (food image task). Motion is measured immediately following functional scans, and the experiment is rerun if motion exceeds 1 mm. A diffusion-weighted scan (DTI) is collected with 37 diffusion directions (FOV = 22 cm, 2.3 mm slices). A high-resolution T1-weighted three-dimensional inversion recovery spoiled gradient-recalled acquisition in the steady state MRI sequence is used (inversion time = 400 ms, RT = 8 ms, echo time = 3.6 ms, flip angle = 151, FOV = 24 cm, 124 slices in coronal plane, matrix = 256 × 192, number of excitations = 2; acquired resolution = 1.5 × 0.9 × 1.1 mm). Images are reconstructed as a 124 × 256 × 256 matrix. We preprocess all data within 1-week of the scan to ensure that all the data are usable. We rescan subjects for whom we have poor data on structural and resting state scans.

#### Task Switching Task

We utilize a hybrid block and event-related design to examine task switching, which has been previously validated (53). This design allows for the separation of neural activity related to the cognitive load of engaging in task switching over a sustained period (block contrast) from the neural activity related specifically to the transient behavior of switching tasks on a particular trial (event-related contrast). Thus, we present blocks of mixed-task trials and blocks of single-task trials. The task requires participants to classify colored shapes (red and blue triangles and circles) based on groupings indicated by a written instruction, such as “Shape” or “Color.” In the mixed-task trials, classification task of the trial are randomly intermixed throughout the block. We present 50 mixed-task trials and 50 single-task trials. This task is delivered in 2 11-min runs, and engagement will be confirmed *via* confirmation of accuracy greater than 80%.

#### Food Image Task

We utilize the same design as in prior literature examining neural response to food images in AN by presenting five blocks of high-calorie food images alternating with five blocks of non-food images (37). Each block consists of 10 images presented for 1.5 s. Participants are instructed to look at each picture attentively. Images are rated after scanning for disgust and fear on a visual analog scale. This task takes approximately 8 min.

#### Risk-taking Task

In addition to the in-scanner tasks, after the scan, participants complete the Angling Risk Task (45) *via* Experiment Factory online (54). During the ART, participants fish for 30 rounds in a pond with different numbers of fish, mostly red fish with one blue fish. Each time the participant selects “Go Fish,” they randomly catch a fish in the pond. Each red fish results in points which can be collected when the participant selects “Collect” and moves on to the next round of fishing. However, if the participant catches the blue fish, the round will end, and the participant will lose all the points earned that round. Participants are told that to keep their points from round to round, they must stop fishing and press “Collect” before they catch a blue fish. There are two tournaments; one in which fish are released back to the pond after being caught, which results in the same number of red and blue fish throughout the round (Catch N Release). In the Catch N Keep tournament, caught fish come out of the lake, thus increasing the chance of catching a blue fish. The points earned in one tournament have no effect on the next, and participants are instructed to try to do as well as possible in both tournaments. Each red fish caught (point) is worth 2 cents, which is added up to a total dollar amount that participants can win at the end of the task (up to $25).

Both this neuroimaging study and the larger RCT have approval from the local Institutional Review Boards.

### Data Analytic Plan

Images will be preprocessed using *fMRIPprep* 1.2.3 (55, 56), which is based on *Nipype* 1.1.6-dev (57, 58).

#### Anatomical Data Preprocessing

All T1-weighted (T1w) will be corrected for intensity non-uniformity (INU) using N4BiasFieldCorrection (59) (ANTs 2.2.0). A T1w-reference map will be computed after registration of all T1w images (after INU-correction) using mri_robust_template [(FreeSurfer 6.0.1, (60)]. The T1w-reference will then be skull-stripped using antsBrainExtraction.sh (ANTs 2.2.0), using OASIS as target template. Brain surfaces will be reconstructed using recon-all [FreeSurfer 6.0.1, (61)], and the brain mask estimated previously will be refined with a custom variation of the method to reconcile ANTs-derived and FreeSurfer-derived segmentations of the cortical gray-matter of Mindboggle (62). Spatial normalization to the ICBM 152 Nonlinear Asymmetrical template version 2009c (Fonov et al., 2009, RRID : SCR_008796) will be performed through nonlinear registration with antsRegistration [ANTs 2.2.0, (63)], using brain-extracted versions of both T1w volume and template. Brain tissue segmentation of cerebrospinal fluid (CSF), white-matter (WM) and gray-matter (GM) will be performed on the brain-extracted T1w using fast [(FSL 5.0.9, (64)].

#### Functional Data Preprocessing

For each BOLD run per subject (across all tasks and sessions), the following preprocessing will be performed. First, a reference volume and its skull-stripped version will be generated using a custom methodology of *fMRIPrep*. The BOLD reference will then be co-registered to the T1w reference using bbregister (FreeSurfer) which implements boundary-based registration (65). Co-registration will be configured with nine degrees of freedom to account for distortions remaining in the BOLD reference. Head-motion parameters with respect to the BOLD reference (transformation matrices, and six corresponding rotation and translation parameters) are estimated before any spatiotemporal filtering using mcflirt [(FSL 5.0.9, (66)]. The BOLD time-series (including slice-timing correction when applied) will be resampled onto their original, native space by applying a single, composite transform to correct for head-motion and susceptibility distortions. These resampled BOLD time-series will be referred to as *preprocessed BOLD in original space*, or just *preprocessed BOLD*. The BOLD time-series are resampled to MNI152NLin2009cAsym standard space, generating a *preprocessed BOLD run in MNI152NLin2009cAsym space*. First, a reference volume and its skull-stripped version are generated using a custom methodology of *fMRIPrep*. Several confounding time-series are calculated based on the *preprocessed BOLD*: framewise displacement (FD), DVARS and three region-wise global signals. FD and DVARS are calculated for each functional run, both using their implementations in *Nipype* [following the definitions by Power and colleagues (67)]. The three global signals are extracted within the CSF, the WM, and the whole-brain masks. Additionally, a set of physiological regressors re extracted to allow for component-based noise correction [*CompCor*, (68)]. Principal components are estimated after high-pass filtering the *preprocessed BOLD* time-series (using a discrete cosine filter with 128s cut-off) for the two *CompCor* variants: temporal (tCompCor) and anatomical (aCompCor). Six tCompCor components are then calculated from the top 5% variable voxels within a mask covering the subcortical regions. This subcortical mask is obtained by heavily eroding the brain mask, which ensures it does not include cortical GM regions. For aCompCor, six components are calculated within the intersection of the aforementioned mask and the union of CSF and WM masks calculated in T1w space, after their projection to the native space of each functional run (using the inverse BOLD-to-T1w transformation). The head-motion estimates calculated in the correction step are also placed within the corresponding confounds file. The BOLD time-series, are resampled to surfaces on the following spaces: *fsaverage5*. All resamplings can be performed with *a single interpolation step* by composing all the pertinent transformations (i.e., head-motion transform matrices, susceptibility distortion correction when available, and co-registrations to anatomical and template spaces). Gridded (volumetric) resamplings will be performed using antsApplyTransforms (ANTs), configured with Lanczos interpolation to minimize the smoothing effects of other kernels (69). Non-gridded (surface) resamplings were performed using mri_vol2surf (FreeSurfer).

Many internal operations of *fMRIPrep* use *Nilearn* 0.4.2 (70), mostly within the functional processing workflow. For more details of the pipeline, see the section corresponding to workflows in *fMRIPrep*’s documentation.

Structural measures of total gray matter volume, white matter volume, cortical surface area, and average cortical thickness will be extracted from FreeSurfer (run during preprocessing). After preprocessing, independent component analysis (ICA) will be conducted using FSL’s MELODIC software to examine intrinsic connectivity networks in r-fMRI. ICA is a statistical technique that separates a set of signals into independent spatiotemporal components (71). It allows for the removal of artifact and the isolation of neural networks (72, 73). We will allow the software to estimate the optimal number of components for each subject. Components will be transformed to standard space, and the DMN and SN will be selected from among each subject’s independent components using an automated template-matching procedure (73). A one-sample t-test will be performed on normalized network maps. These maps will be used to generate connectivity scores for each ICN.

Condition effects for the task-based fMRI will be estimated using the general linear model. The response to events will be modeled by a canonical hemodynamic response function (HRF). The task switching task has two events of interest: task-switch events and task-repeat events contrasted with each other, as well as two blocked conditions: mixed-task blocks and single-task blocks in another contrast. The food image task has two events of interest: food images and non-food images contrasted with each other. Condition-specific estimates of neural activity (betas, corresponding to the height of the HRF) will be computed independently at each voxel for each subject. Analyses will be conducted in FSL FEAT, which utilizes cluster-based thresholding and Gaussian Random Field (GRF) theory to identify significant clusters of voxels for each analysis. Although we have hypotheses related to certain brain regions, we will conduct whole brain analyses to ensure we are not biasing results.

DTI data will be analyzed with FSL Diffusion Toolbox (FDT) to perform affine registration to correct for eddy current distortions, extract brain from skull in the image, and fit tensors and tracts to the data. Further tensor fitting and fiber tracing will be completed with Diffusion Toolkit software. Primary measures will be of fractional anisotropy (the degree to which diffusion takes place in a given direction) and R1 (measure of relaxation rates of water protons, which has a strong relation with myelin content). Probabilistic tractography will be explored using FSL’s built-in tools, particularly along fronto-striatal pathways, which may be impacted in AN (74). Of note, DTI data, as well as structural data, can be impacted by effects of dehydration, which may relate to malnutrition (75). Although objectively assessing hydration status specifically would be ideal, it is not possible in this small study. However, we will include reports of hydration, as well as recent hospitalizations, as many of the patients in the trial were in the hospital for medical stabilization (including hydration) prior to enrolling in the study.

Behavioral data from the risk-taking task will be examined by calculating the adjusted score, which is the average number of casts taken on rounds when participants chose to stop fishing. Higher scores indicate greater risk-taking.

Across all measures (gray and white matter volume, cortical surface area, cortical thickness, r-fMRI connectivity for SN and DMN, DTI FA and R1, task-based contrasts, and risk-taking scores), we will examine change over time using repeated measures analyses, including mixed effects modeling for extracted scores or repeated measures modeling in FSL for task-based measures. Because EOT will occur at different times after baseline depending on treatment duration, time will be included as a covariate. Primary comparisons will be conducted between those who gain 2.4 kg by session 4 and those who do not (early responders vs. non-early responders). We will also use weight change as a continuous variable to examine how change in the brain and behavior measures is associated with weight change, particularly if we have small cell size in one of the two responder groups. Exploratory analyses will also examine baseline brain and behavior measures as predictors of early response and end of treatment outcome *via* mixed effects.

## Discussion

Studies of neural change over the course of treatment in anorexia nervosa, particularly early in treatment, have the potential to increase motivation for early refeeding behavior among parents of adolescents. A recently developed treatment incorporating neurobiological research findings into treatment was considered helpful in improving understanding of AN (76), suggesting that improving knowledge about neurobiological changes through treatment may be of interest to patients and families. Anecdotally, we often hear parents and clinicians express concern about not directly targeting eating disorder cognitions early in treatment. There is worry that focusing on refeeding ignores the emotional needs of the patient. Evidence of neural change early in FBT could alleviate these concerns by showing how cognitive and neurobiological functions can be impacted without being specific targets of treatment. Further, while brain plasticity during adolescence may create a vulnerability under which AN develops (77), it can also allow for greater opportunities for neural changes (78), providing some explanation why AN that is not treated during adolescence may become enduring (79).

Conducting neuroimaging research in the context of treatment for adolescent AN comes with a number of challenges. First, families are often anxious when first receiving a diagnosis and report feeling overwhelmed by deciding on treatment. It can be difficult for families to agree to participate in a study that does not have an immediate, tangible benefit for their child’s care. Later in recovery, we find that families may be more open to neuroimaging research, but at that point, we can no longer obtain pre-treatment data. Second, the timing of scans can create challenges in obtaining complete data for all participants. Families often, understandably, want to begin treatment as soon as possible, so scheduling a pre-treatment scan can feel like a delay of treatment. Our team often conducts scans on the same day as and just prior to session 1 in order to assuage this concern. Follow-up scans are also challenging to schedule, particularly the early treatment timepoint because there is a short window of time to complete the scan. Illnesses, busy schedules for scanners, or technical problems with the scanner can impact the collection of these data. Our team addresses this by scheduling the early response scan as early as possible so that it is on everyone’s schedule in advance, and our research coordinators are in constant communication with families to ensure that the timeline will still work. Scheduling as early in the time window as possible also allows for some buffer to reschedule in case of illness or technical problem. Finally, there may be a bias in samples for neuroimaging studies due to mistrust of research (80), anxiety about adding too much to the child’s schedule, or other reasons for declining participation (81). Although some of these biases may be exacerbated in neuroimaging studies of treatment effects due to the additional burden of assessment and treatment sessions being scheduled simultaneously, they may also be true of neuroimaging studies broadly. Thus, generalization of results must be made cautiously.

Overall, investigating brain and behavior changes over the course of FBT has great potential to improve our understanding of early treatment response and motivate engagement in treatment from the start. While many of the most pronounced neural differences in AN seem to recover with weight restoration, knowledge about how early these changes occur can support timing of treatment interventions. Further, a greater understanding that specific interventions targeting cognitive processes may not be necessary for neural and cognitive change can address fears that some families express about engaging in FBT. If hypotheses are supported from this preliminary study and in later confirmatory studies, future research could examine the influence of education about neurobiological change on treatment engagement. Further, if baseline brain function is predictive of treatment response, future research could consider augmentations or alternative treatments for those patients at high-risk for non-response.

## Data Availability Statement

The dataset for this study will be available upon study completion.

## Ethics Statement

This protocol was approved by the IRB at Stanford University. Written informed consent will be obtained from all participants.

## Author Contributions

The design of the study was developed through collaboration between all three authors. NW contributed to task selection for the protocol. CB wrote the manuscript, and NW and JL edited the text.

## Funding

The study is funded by a grant to CB from the Hilda and Preston Davis Foundation.

## Conflict of Interest

The authors declare that the research was conducted in the absence of any commercial or financial relationships that could be construed as a potential conflict of interest.
